# Supplementary material for: Deletion of wfs1 Impairs Oligodendrocyte Precursor Cells Dorsal Distribution and Myelination Through the wfs1-hmgcs1 Axis in Zebrafish
Source: Biology (Basel). 2026 Aug 16;15(16):1402. doi: 10.3390/biology15161402 (PMC13510151; doi:10.3390/biology15161402)
Supplement: Supplementary file 1 [file biology-15-01402-s001.zip › File S1-Transcriptome sequencing data and differentially expressed genes (DEGs) in wfs1-- zebrafish at 5 dpf/1.GO/wfs1KOvsWT/ALL/wfs1KOvsWT.all_GObar_mf.pdf]

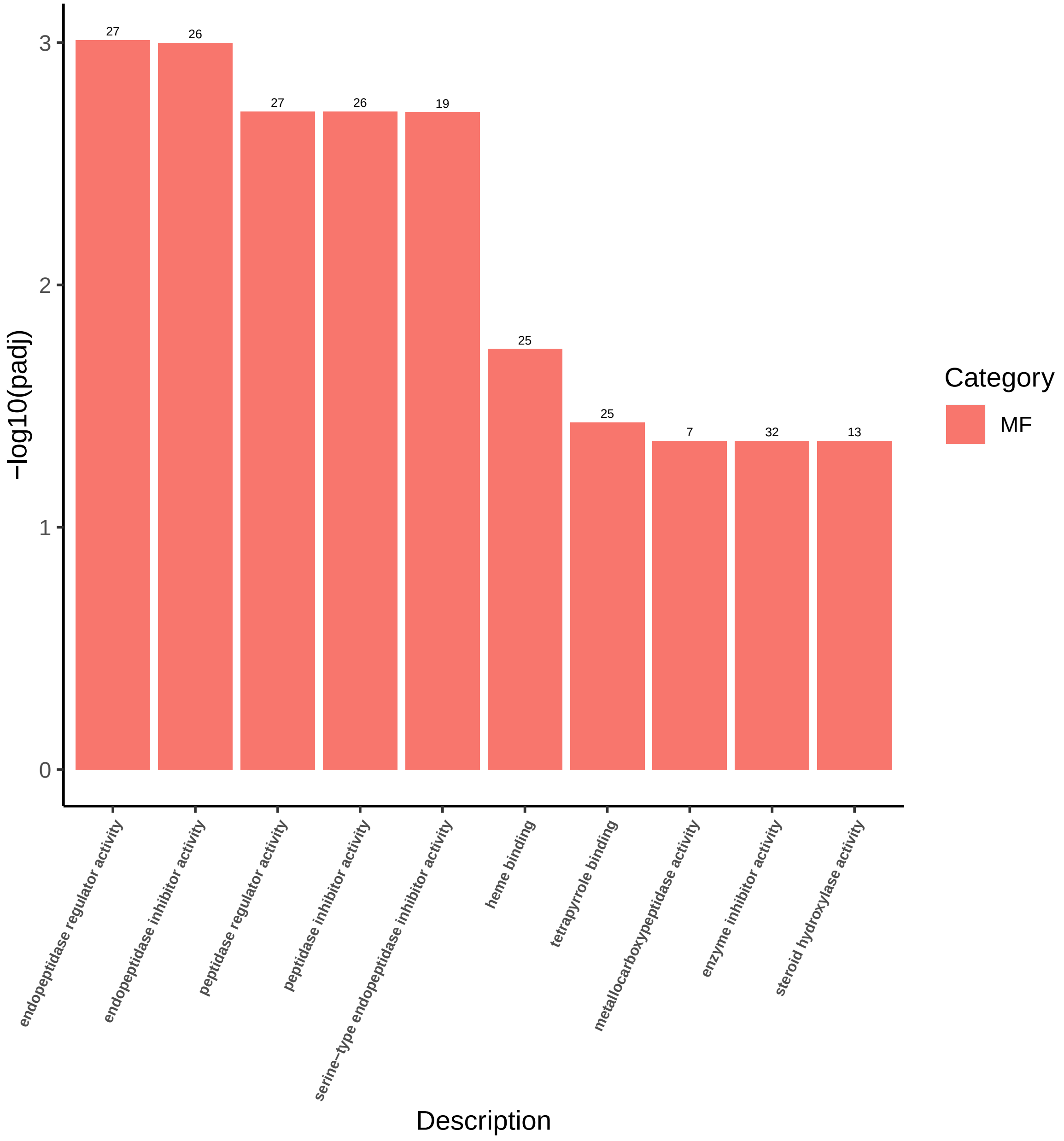
27
26
27
26
19
25
25
7
32
13
3
2
1
0
−log10(padj)
endopeptidase regulator activity
endopeptidase inhibitor activity
peptidase regulator activity
peptidase inhibitor activity
serine−type endopeptidase inhibitor activity
heme binding
tetrapyrrole binding
metallocarboxypeptidase activity
enzyme inhibitor activity
steroid hydroxylase activity
Description
Category
MF
